# Data-driven *p*-norms for estimating transmission loss coefficients in power systems

**DOI:** 10.1371/journal.pone.0345033

**Published:** 2026-03-18

**Authors:** Oscar Danilo Montoya, Walter Gil-González, Luis Fernando Grisales-Noreña

**Affiliations:** 1 Grupo de Compatibilidad e Interferencia Electromagnética (GCEM), Facultad de Ingeniería, Universidad Distrital Francisco José de Caldas, Bogotá, Colombia; 2 Department of Electrical Engineering, Faculty of Engineering, Universidad Tecnológica de Pereira, Pereira, Colombia; 3 Grupo de Investigación en Alta Tensión—GRALTA, Escuela de Ingeniería Eléctrica y Electrónica, Facultad de Ingeniería, Universidad del Valle, Cali, Colombia; Aalto University, FINLAND

## Abstract

This research introduces a novel convex methodology for estimating transmission loss coefficients (*B*-coefficients) in power systems using a data-driven approach based on power system measurements. To enhance estimation accuracy and practical relevance, the model is evaluated across a wide spectrum of operating conditions, incorporating random variations in active power injections and demand profiles modeled via uniform and Gaussian distributions. A semi-definite programming (SDP) model leveraging *p*-norm formulations is proposed to derive the *B*-coefficients efficiently. Numerical evaluations on IEEE 14-, 39-, 57-, and 118-bus test feeders demonstrate the effectiveness and robustness of the approach, yielding average estimation errors between −6% and 5% across diverse scenarios. These results confirm the reliability of the proposed methodology, contributing to improved accuracy in transmission loss modeling and supporting more efficient power system operations.

## 1 Introduction

Optimizing generation scheduling in electrical systems is vital for ensuring that the energy dispatch complies with constraints while minimizing costs [1]. Achieving an optimal economic dispatch (OED) is crucial for the operational efficiency and reliability of power systems, as it aids in identifying the best combination of generation units to meet the demand while minimizing operational expenses [2]. Key to OED is the accurate estimation of transmission loss coefficients, or *B*-coefficients, which represent the losses in the transmission network [3].

Given the importance of *B*-coefficients in coordinating power plants within transmission networks, this research extends their estimation through semi-definite programming (SDP) via general *p*-norm objective functions. A practical methodology is presented which utilizes data from power system measurements and recursive power flow simulations, employing optimization theories and convex modeling frameworks to address uncertainty in demand profiles. The choice of SDP with *p*-norms as the optimization strategy has a significant impact on ensuring optimal solutions, enhancing the robustness of the estimation technique [4].

While traditional estimation frameworks focus primarily on predictive accuracy, recent research has emphasized cost-oriented forecasting, where prediction models are optimized to directly improve downstream decision-making performance (e.g., economic dispatch or unit commitment) rather than solely minimizing statistical error metrics. This aligns with the closed-loop predict-and-optimize paradigm, which integrates forecasting with operational optimization to enhance overall system economics [5]. In this context, our proposed SDP framework for *B*-coefficient estimation provides a natural foundation for extension toward cost-aware learning. By embedding the loss coefficient model within a bilevel or decision-focused optimization structure, future work could adapt the estimation process to minimize not only prediction errors, but also the resulting operational costs, thereby bridging accuracy-oriented estimation with cost-driven performance in power system dispatch [6].

The estimation of *B*-coefficients has been a subject of considerable research. Early works, such as [7], introduced Kron reduction for efficient determination, while [8] applied the least squares method, effectively capturing fluctuations in power losses through a quadratic formulation. Notably, [9] used genetic algorithms for estimating *B*-coefficients in dynamic dispatch contexts. Moreover, both [10] and [11] employed least-squares optimization methods for estimating *B*-coefficients, emphasizing the significance of grid topological data in obtaining accurate results [12].

Recent advancements in estimation methods have increasingly utilized measurement-based approaches, which adapt to variable generation inputs and demand profiles, often leveraging high-resolution data from devices such as phasor measurement units (PMUs). Notably, [3] employed a semidefinite programming (SDP) formulation for estimating power loss coefficients, reporting errors below 3.5% in numerical evaluations on IEEE test systems. Building upon this foundation, our work extends the SDP framework to a more general convex model that incorporates *p*-norm objective functions. This generalization allows the estimator to accommodate a range of error sensitivities and enhances robustness to the inherent variability and uncertainty present in generation and demand patterns.

This work enhances the accuracy of loss estimation as well as operational efficiency, offering an innovative methodology for estimating *B*-coefficients in power systems. The convex optimization model not only computes *B*-coefficients but also ensures that associated quadratic loss coefficients conform to a positive-semidefinite symmetric matrix, thereby maintaining physical realism. The adaptability of our methodology enables parameter recalibration in response to changes in topology or configurations, ultimately improving reliability and efficiency in power system operations.

The remainder of this document is structured as follows. Section 1 outlines the theoretical foundations of *p*-norms by presenting their general formulation along with some of the most recognized cases. Section 2 formulates a general optimization problem for estimating *B*-coefficients in power systems, considering measurement data through quadratic representations. Section 3 discusses the SDP formulation and highlights the key elements necessary to understand this convex optimization model, as proposed by the authors in [3], demonstrating how this model can be generalized using a *p*-norm representation, which is elaborated upon in Section 4. Numerical validations across different IEEE test systems are examined in Section 5, focusing on some of the most relevant cases of the *p*-norms. Finally, Section 6 presents the main conclusions drawn from this research and explores potential future directions.

## 2 Theoretical foundations of *p*-norms

The *p*-norm, also known as the $\ell^{p}$ *norm*, is a fundamental concept in functional analysis and vector space theory, providing a means to measure the magnitude of vectors in ${\mathbb{R}}^{n}$ or ${\mathbb{C}}^{n}$ [13]. Formally, for a vector $x=(x_{1},x_{2},\ldots ,x_{n})$, the *p*-norm is defined as follows:


$$\|x{\|}_{p}={\left(\sum_{i=1}^{n}|x_{i}{|}^{p}\right)}^{1/p},$$ (1)

where p≥1. This family of norms satisfies the properties of positivity, scalability, triangle inequality, and definiteness, positioning it as a valid metric for the vector space [14]. Notably, as p→∞, the *p*-norm converges to the supremum norm:


$$\|x{\|}_{\infty }=\max_{1\leq i\leq n}|x_{i}|,$$ (2)

which measures the largest component in magnitude. The choice of *p* influences geometric properties and robustness: while the Euclidean norm (p=2) is associated with classical geometric interpretations, the $\ell^{1}$ norm emphasizes sparsity and robustness to outliers, and the $\ell^{\infty }$ norm focuses on worst-case deviations. These properties make *p*- norms versatile tools in areas such as optimization, statistical inference, and signal processing, where they facilitate diverse metrics tailored to specific analytical needs [15].

## 3 Problem formulation

The objective of the base quadratic method (*B* coefficients) is to approximate the total active power losses as a quadratic function of the generated power $P_{g}$, which is expressed as follows [3]:


$$P_{L}({𝐏}_{G})={𝐏}_{G}^{T}{𝐁}_{20}{𝐏}_{G}+B_{10}^{T}{𝐏}_{G}+B_{00},$$ (3)

where ${𝐁}_{20}\in {\mathbb{R}}^{n\times n}$ is a symmetric and positive-semidefinite matrix responsible for capturing the quadratic effects and interactions between generators. The vector $B_{10}\in {\mathbb{R}}^{n}$ contains linear coefficients associated with each generator, and $B_{00}\in \mathbb{R}$ is a constant coefficient representing the no-load losses of the system. The vector ${𝐏}_{G}$ represents a matrix of generated powers, with *m* measurements for *n* generators, while $P_{L}$ denotes the vector of observed power losses, calculated as the cumulative resistive losses across transmission lines and transformers in the system.

The formulation presented in (3) is particularly advantageous since it allows for a precise and efficient estimation of active losses in the network, without the need to solve a complete power flow analysis [16]. This attribute facilitates real-time operational decision-making and optimizes system management, a crucial capability in environments where agility and accuracy in loss estimations are paramount [17].

**Remark 1** *In a power system characterized by multiple power measurements (i.e., generation and demand), the effective characterization of B-coefficients can be formulated as an optimization problem [8], as demonstrated in Equation (4):*


$$\min E_{m}=\left|{\bar{P}}_{L}-P_{L}\right|,$$ (4)

*where* $E_{m}$ *represents the estimation error,* $P_{L}$ *denotes the calculated power losses (see* Equation (3)*), and* ${\bar{P}}_{L}$ *is the vector of available measurements. The authors of [11] proposed a measurement-based approach for systems with* $N_{g}=|𝐆|$ *generators, necessitating a number of measurements* $m=\frac{N_{g}(N_{g}+3)}{2}+1$*—this holds under the assumption that the* $B_{20}$ *matrix is symmetric. Given this number of measurements, and considering that the optimization model in* Equation (4) *reaches a global optimum when* $E_{m}^{⋆}=0$*, this methodology ensures accurate parameter estimation.*

Note that the minimum measurement requirement stems directly from the total number of independent unknowns in the quadratic loss model. Specifically:

- The symmetric matrix $B_{20}$ has $\frac{N_{g}(N_{g}+1)}{2}$ unique entries,
- The linear coefficient vector $B_{10}$ adds $N_{g}$ parameters,
- The constant term $B_{00}$ contributes one additional scalar.

Thus, the total number of parameters to be estimated is $m=\frac{N_{g}(N_{g}+1)}{2}+N_{g}+1=\frac{N_{g}(N_{g}+3)}{2}+1$. At least this many linearly independent measurement scenarios are required for the estimation problem to be well-posed.

Next, the SDP model proposed by the authors of [3] is presented, along with the model generalization using p−norms.

## 4 SDP model

In this initial model, the proposed formulation employs SDP to overcome the limitations of traditional linear methods. As noted by [3], this convex optimization model allows calculating *B* coefficient while incorporating constraints ensuring that the coefficients associated with quadratic losses form a symmetric and positive-semidefinite matrix. In this vein, the following mathematical formulation is presented:


$$\text{O.F.. }\min_{B_{20},\;B_{10},\;B_{00}}E_{m}=\sum_{m\in ℳ}\left|P_{L_{m}}-{\hat{P}}_{L_{m}}\right|,$$ (5)

$$\text{S.t.: }B_{20}^{T}-B_{20}=0,$$ (6)

$$B_{20}⪰0,$$ (7)

$$P_{L_{m}}=\left(P_{G_{m}}^{T}B_{20}P_{G_{m}}\right)+B_{10}^{T}P_{G_{m}}+B_{00},\;(m\in ℳ),$$ (8)

$$\text{diag}(B_{20})\geq 0,$$ (9)

$$\alpha \leq B_{00}\leq \beta .$$ (10)

The variables in this formulation represent various aspects of the loss estimation model: ℳ denotes the set of available measurements within the system, where each element corresponds to a specific measurement—such as the losses $P_{L_{m}}$, which are those assessed in the system for the measurement *m*, and ${\hat{P}}_{L_{m}}$, *i.e.*, the estimated losses obtained via the convex model. The vector $P_{G_{m}}$ corresponds to the generation power associated with measurement *m*. The quadratic loss coefficients are represented by the matrix $B_{20}$, which, under the constraint $B_{20}^{T}-B_{20}=0$, is guaranteed to be symmetric, while the condition $B_{20}⪰0$ ensures that it is positive-semidefinite, making sure that the losses do not become negative with increases in generation. Additionally, $\text{diag}(B_{20})\geq 0$ imposes that the self-loss terms are non-negative. The vector $B_{10}$ encompasses the linear coefficients, and the coefficient $B_{00}$ models the constant, generation-independent component of total system losses. Physically, it represents *no-load losses* such as transformer core losses (hysteresis and eddy currents), standing corona losses on transmission lines, auxiliary power in substations, and other parasitic loads present even at zero generation dispatch. The bounds $\alpha \leq B_{00}\leq \beta$ are introduced to ensure physical realism during parameter estimation. The lower bound α≥0 reflects the fact that constant losses cannot be negative in a passive network. The upper bound *β* is based on the aggregate rated no-load losses of system components, preventing the convex fitting from attributing excessive loss to the constant term, which could distort the quadratic and linear coefficients ($B_{20}$ and $B_{10}$). These constraints thus incorporate prior engineering knowledge, stabilize the numerical estimation, and improve model extrapolation beyond the measured operating points.

## 5 Generalization to the *p*-norm model

This section extends the framework for estimating transmission loss coefficients by employing the concept of *p-norms*. A *p*-norm generalizes the measurement of vector magnitudes in power systems and is defined as follows:


$$\|\Delta P{\|}_{p}={\left(\sum_{m\in ℳ}{\left|P_{L_{m}}-{\hat{P}}_{L_{m}}\right|}^{p}\right)}^{1/p},$$ (11)

The parameter *p* allows for flexibility in defining the objective function, capturing various aspects of loss sensitivity depending on its value. For instance, using p=2 emphasizes the minimization of mean square errors, whereas p=1 focuses on minimizing the sum of absolute deviations, and p=∞ targets the worst-case scenario regarding losses. The intermediate case p=3 applies a cubic penalty, placing stronger emphasis on moderate to large errors than p=2 without being dominated solely by the maximum error as in the $\ell^{\infty }$-norm. This provides a balanced trade-off, encouraging the model to reduce significant estimation mismatches that are critical for economic dispatch, while maintaining convexity and computational tractability within the SDP framework.

This generalized *p*-norm model facilitates the formulation of a robust optimization problem for estimating the coefficients associated with transmission losses. By applying *p*-norms, the model can be tailored to specific operating conditions and loss characteristics, enhancing the accuracy of loss estimations and improving the overall management efficiency of the system.

**Remark 2** *The case of p=1, i.e., $\ell^{1}$-norm, corresponds to the SDP formulation presented by the authors of [3].*

**Fig 1:**
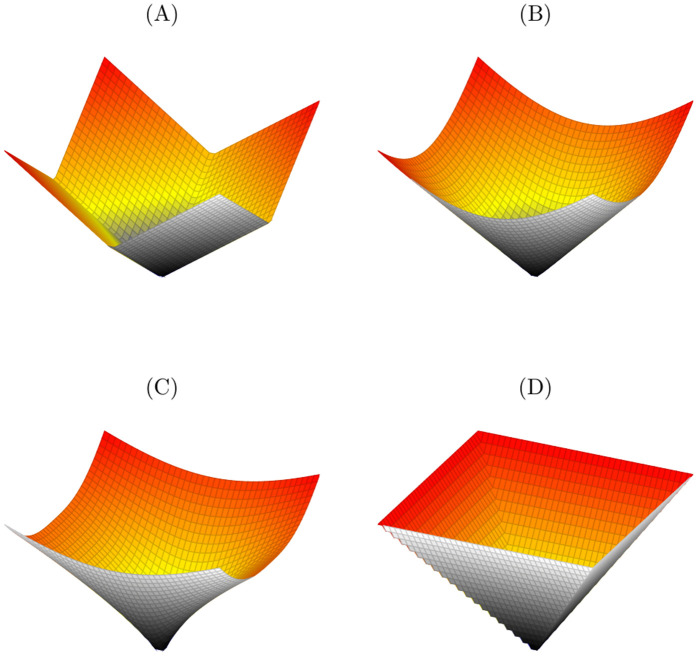
Graphical representation of the $\ell^{p}$-norms: **A)** **$\ell^{1}$-norm, B) $\ell^{2}$-norm,** **A)** **$\ell^{3}$-norm,** **D)** **$\ell^{\infty }$-norm**.

Fig 1 illustrates the diverse geometric shapes of the $\ell^{p}$-norms in a three-dimensional space, showcasing how each norm defines a distinct measure of distance. The $\ell^{1}$-norm in 1a is represented by a diamond-like shape, reflecting the sum of absolute values of the coordinates and highlighting equal contributions along each axis. The $\ell^{2}$-norm in 1b is depicted as a spherical surface, embodying the familiar Euclidean distance. The $\ell^{3}$-norm in 1c exhibits a more complex configuration, demonstrating its usefulness in generalized distance calculations. Finally, the $\ell^{\infty }$-norm in 1d is characterized by a cubic form that emphasizes the worst-case scenario of dimensional displacement. Together, these visualizations underscore the unique mathematical properties of the norms, as well as their applicability in modeling and optimization contexts.

The optimization model based on *p*-norms combines the general objective function in (11) with the physical constraints of the SDP formulation from (6) to (10). For completeness, the proposed formulation to estimate the transmission loss coefficients is presented as follows:


$$\begin{array}{cc} \text{O.F. } & \min_{B_{20},\;B_{10},\;B_{00}}E_{m}={\left(\sum_{m\in ℳ}{\left|P_{L_{m}}-{\hat{P}}_{L_{m}}\right|}^{p}\right)}^{1/p},\\ \text{S.t.: } & B_{20}^{T}-B_{20}=0,\\ & B_{20}⪰0,\\ & P_{L_{m}}=P_{G_{m}}^{T}B_{20}P_{G_{m}}+B_{10}^{T}P_{G_{m}}+B_{00},\;m\in ℳ,\\ & \mathrm{diag}(B_{20})\geq 0,\\ & \alpha \leq B_{00}\leq \beta .\; \end{array}$$ (12)

**Remark 3** *The structure of model (12) remains convex and belongs to the family of SDP problems, since the positive semidefinite constraint $B_{20}⪰0$ is preserved. This ensures that the quadratic loss term is physically meaningful and that the overall optimization problem can be solved efficiently using convex solvers.*

## 6 Numerical validations

The performance of the proposed Optimization Model (12) was evaluated using 14-, 39-, 57-, and 118-bus test systems. Four objective functions were considered, namely the linear, $\ell^{1}$-, $\ell^{2}$-, $\ell^{3}$-, and $\ell^{\infty }$-norm formulations. For each test system, a comprehensive training dataset comprising 𝐌 operating scenarios—with 𝐌 deliberately exceeding the minimum theoretical requirement of $N_{g}(N_{g}+3)/2+1$—was synthetically generated. To capture a wide spectrum of realistic operating conditions, the generator active power outputs were varied using both uniform and Gaussian distributions within a range of 50% to 100% of their nominal capacities. Simultaneously, load demands were independently perturbed following the same probabilistic approaches, with values spanning from 40% to 100% of their nominal magnitudes. This dual-distribution sampling strategy ensures that the training set encapsulates both bounded uniform variability and normally distributed fluctuations, thereby enhancing the statistical richness of the data and the generalizability of the estimated coefficients. These perturbation ranges were carefully selected to realistically emulate the operational variability commonly encountered in practical power systems [18]. All numerical experiments were conducted using MATPOWER 8.1 within the MATLAB R2025b environment, and the optimization problems were implemented in YALMIP and solved using the MOSEK solver [19]. All optimization problems were solved using the solver’s default optimality tolerances, requiring no additional parameter tuning. To ensure full reproducibility and transparency, all simulation codes and datasets have been made publicly available [20].

To ensure full reproducibility of the results, all scripts and datasets used in this study are publicly available in an open-access GitHub repository available in [20].

To evaluate the *out-of-sample* generalization capability of each fitted model, an additional validation dataset of 1000 random operating scenarios was generated under the same operating conditions. These validation points were not used during the optimization process and serve solely to assess the predictive accuracy of the estimated loss coefficients across unseen operating states.

**Fig 2:**
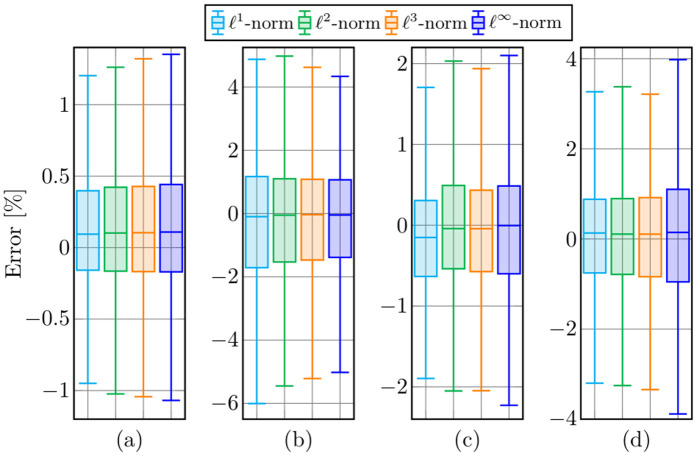
Distribution of estimation errors represented by boxplots: A) the 14-bus test system, B) the 39-bus test system, C) the 57-bus test system, and D) the 118-bus test system. The box denotes the interquartile range (Q1–Q3), the horizontal line indicates the median, and the whiskers represent the minimum and maximum values.

Fig 2 illustrates the boxplots obtained for each objective function under evaluation. This figure summarizes the distribution of estimation errors for all test systems, including their median, minimum, and maximum values as well as the first- and third-quartile ranges. The results for the SDP model are not included, since this formulation is equivalent to the $\ell^{1}$-norm model; both yield identical outcomes.

From Fig 2, the following can be stated:

- In the 14-bus test system, the estimation errors remain very small for all norms, with values roughly ranging from −1.1% to 1.3% (see Fig 2A). The interquartile ranges for all norms are approximately within [−0.2%,0.4%], indicating high consistency among the studied methods. The median errors remain close to zero for all norms. However, for this test system, the proposed $\ell^{1}$-norm model shows a better performance.
- In the 39-bus test system, the errors range approximately from −6% to 5% (see Fig 2B), showing higher variability compared to the 14-bus system. The $\ell^{\infty }$-norm shows the smallest quartile ranges, ≤10% than other norms. All median values remain close to zero, which indicates proper estimations. Although variability increases with system size, the ℓ∞2 model provides the most stable performance among the evaluated norms.
- In the 59-bus test system, the estimation errors lie roughly between −2.3% and 2.2% (see Fig 2B), and the interquartile ranges of the four norms fall within [−0.6%,0.5%], smaller in comparison with the 39-bus feeder. In addition, for the 59-bus test system, the $\ell^{1}$-norm shows a slightly larger dispersion compared to the other norms, whose medians remain almost centered at zero.
- The 118-bus test system exhibits an increase in the estimation error range: approximately from −4% to 4%. The $\ell^{\infty }$-norm shows a slightly greater upper spread, while the $\ell^{2}$- and $\ell^{3}$-norms maintain the smallest median deviations (close to zero). These results confirm that the proposed model scales effectively, preserving a narrow error distribution even in large-scale grids.

Overall, the numerical results demonstrate the high accuracy and robustness of the proposed optimization models across all test systems. In every case, 50% of the estimation errors lie within a narrow interval, ranging approximately from −1.5% to 1.1% in the worst scenario (see Fig 2B), indicating that the proposed formulations consistently deliver low-dispersion estimations. Moreover, the mean errors remain close to zero for all norms and system sizes, confirming that the proposed $\ell^{p}$-based models do not systematically overestimate or underestimate the power losses. These findings highlight the effectiveness of the proposed approach in providing reliable loss approximations under diverse operating conditions and system scales.

Table 1 summarizes the numerical performance of the proposed $\ell^{p}$-based models across the four test systems. The reported metrics include the mean percentage estimation error, the standard deviation, and the interquartile range (IQR) of the error evaluated over 1000 operating scenarios, as well as the computational time required to estimate the corresponding *B*-coefficients.

**Table 1:** Comparison of the performance of the proposed $\ell^{p}$-based models.

Nodes	$N_{g}$	Model	Mean [%]	Std [%]	IQR [%]	Time [s]
14	5	ℓ_1_	-0.0332	0.1065	0.1119	0.2175
		ℓ_2_	-0.0371	0.0915	0.1015	0.3191
		ℓ_3_	-0.0361	0.0869	0.1030	0.4025
		ℓ_∞_	-0.0359	0.1010	0.1257	0.2448
39	10	ℓ_1_	-0.1135	1.2181	1.2403	0.2946
		ℓ_2_	-0.0583	1.1125	1.1483	0.2969
		ℓ_3_	-0.0597	1.0707	1.1420	0.7258
		ℓ_∞_	-0.1711	1.0381	1.1167	0.3192
57	7	ℓ_1_	-0.2867	0.8441	1.0399	0.2564
		ℓ_2_	-0.1803	0.7232	0.8707	0.2819
		ℓ_3_	-0.1782	0.7306	0.8546	0.5757
		ℓ_∞_	-0.2309	0.7480	0.9038	0.2787
118	54	ℓ_1_	-0.0110	0.2059	0.2533	5.8629
		ℓ_2_	-0.0118	0.1807	0.2246	5.1325
		ℓ_3_	-0.0117	0.1812	0.2330	15.6600
		ℓ_∞_	-0.0128	0.1880	0.2501	8.8000

From Table 1, it can analyze that:

- For 14-bus test system, all $\ell^{p}$-based models exhibit very small mean estimation errors, close to zero, along with low standard deviations and IQRs. This indicates a high level of accuracy and consistency in loss estimation under relatively simple network conditions. The $\ell_{2}$- and $\ell_{3}$-norm formulations achieve the lowest dispersion, while the $\ell_{1}$-norm provides comparable accuracy with slightly reduced computational time. Overall, all models perform reliably in small-scale systems.
- In 39-bus test system, a moderate growth in estimation variability is observed across all norms. Nevertheless, the mean errors remain close to zero, confirming the absence of systematic bias. The $\ell_{\infty }$-norm achieves the smallest standard deviation and IQR, indicating superior robustness against extreme estimation errors in this medium-scale system. However, this robustness is obtained at the expense of higher computational effort compared to the $\ell_{1}$- and $\ell_{2}$-norm formulations.
- For the 57-bus system, the proposed models maintain low mean errors and moderate dispersion levels, demonstrating good scalability. The $\ell_{2}$- and $\ell_{3}$-norms yield the smallest standard deviations and IQR values, suggesting a balanced trade-off between robustness and accuracy. In contrast, the $\ell_{1}$-norm exhibits slightly larger dispersion, reflecting higher sensitivity to operating-point variability in this test case.
- In the large-scale 118-bus system, all $\ell^{p}$-norm models preserve mean errors extremely close to zero, confirming their unbiased behavior even under high-dimensional conditions. Although the dispersion metrics remain low, the computational time increases significantly due to the higher number of generators and decision variables. Among the evaluated norms, the $\ell_{2}$-norm offers the best compromise between accuracy, robustness, and computational efficiency, whereas the $\ell_{3}$-norm incurs the highest computational burden with marginal accuracy improvements.

Overall, Table 1 shows that the $\ell_{3}$-norm formulation consistently requires the longest computational time across all test systems, with an average increase of approximately 40–60% compared to the $\ell_{1}$- and $\ell_{2}$-norm models, and even higher in large-scale systems. This behavior is associated with the higher-order nonlinearity of the cubic penalty, which increases the solver complexity. However, the $\ell_{3}$-norm generally achieves smaller IQRs in most test systems. This means that this norm concentrates the estimation errors around the median and improved robustness under typical operating conditions. Therefore, the $\ell_{3}$-norm offers enhanced estimation stability at the expense of increased computational effort, making it more suitable for offline or planning-oriented applications.

On the other hand, in Table 1 can be also noted that the 39-bus test system exhibits higher estimation dispersion and larger error ranges compared to the other test systems. This behavior can be explained by its structural and operational characteristics.

## 7 Conclusions

This study extended the estimation of transmission loss coefficients (*B*-coefficients) in electrical power systems through a convex SDP framework leveraging general $\ell^{p}$-norm objective functions. The proposed formulations preserve the physical consistency of the quadratic loss model while enhancing robustness against operating-point variability in generation and demand. Numerical results obtained from several IEEE benchmark systems demonstrated that all $\ell^{p}$-based models achieve low-dispersion estimation errors with mean values close to zero, confirming their unbiased behavior across diverse operating scenarios. In particular, the comparative analysis revealed a clear trade-off between computational efficiency and robustness: while the $\ell_{1}$- and $\ell_{2}$-norm formulations provide faster solutions, the $\ell_{3}$-norm consistently yields tighter interquartile ranges, indicating improved stability of loss estimates under typical operating conditions. These findings highlight the flexibility of the proposed framework, allowing practitioners to select the most suitable norm depending on accuracy, robustness, and computational requirements. Moreover, the methodology exhibits strong adaptability, as the data-driven estimation process can be readily updated to accommodate changes in system topology or operating conditions without requiring explicit network parameter identification. This feature is particularly valuable for modern power systems with increasing variability and uncertainty, supporting more reliable economic dispatch and operational planning.

Future research will focus on several interconnected directions to extend the applicability and performance of the proposed methodology. First, learning-based techniques will be explored to enhance estimation accuracy under highly dynamic operating conditions, particularly by incorporating real-time measurements from PMUs. A key methodological challenge lies in bridging the gap between high-frequency PMU data (typically 30–60 samples per second) and the current 15-minute measurement framework. Potential integration pathways include statistical aggregation of PMU data over each interval to extract meaningful features (e.g., means, variances, or extreme events) that can serve as additional inputs to the estimation model, as well as developing recursive updating schemes that enable near-real-time coefficient adaptation using streaming measurements. Second, a systematic comparative analysis between the proposed convex SDP framework and modern non-convex machine learning approaches, such as artificial neural networks, will be undertaken for transmission loss coefficient estimation. While the convex formulation guarantees global optimality, physical interpretability (e.g., positive semidefiniteness), and computational efficiency, data-driven techniques offer greater flexibility to capture complex non-linear relationships without relying on predefined quadratic structures. A comprehensive benchmark study evaluating estimation accuracy, generalization under noisy data, computational performance, and physical constraint adherence would contextualize the advantages of each approach and could guide the development of hybrid strategies that leverage the robustness of convex optimization alongside the representational power of machine learning. Finally, extending the proposed $\ell^{p}$-norm formulations to larger-scale systems and validating their performance using real-world field data constitute essential steps toward improving the resilience and efficiency of practical power system operations, with the ultimate goal of delivering tailored solutions based on specific application requirements and data availability.
